# How to Better Integrate Social Determinants of Health into Primary Healthcare: Various Stakeholders’ Perspectives

**DOI:** 10.3390/ijerph192315495

**Published:** 2022-11-22

**Authors:** Catherine Hudon, Olivier Dumont-Samson, Mylaine Breton, Yann Bourgueil, Christine Cohidon, Hector Falcoff, Nicolas Senn, Thérèse Van Durme, Émilie Angrignon-Girouard, Sarah Ouadfel

**Affiliations:** 1 Département de Médecine de Famille et de Médecine D’urgence, Université de Sherbrooke, Sherbrooke, QC J1H 5N4, Canada; 2 Centre de Recherche du Centre Hospitalier Universitaire de Sherbrooke, Sherbrooke, QC J1H 5N4, Canada; 3 Département des Sciences de la Santé Communautaire, Université de Sherbrooke, Longueuil, QC J4K 0A8, Canada; 4 Chaire Santé Sciences Po, 75007 Paris, France; 5 Département de Médecine de Famille, Centre Universitaire de Médecine Générale et Santé Publique (Unisanté), Université de Lausanne, 1011 Lausanne, Switzerland; 6 Société de Formation Thérapeutique du Généraliste/Recherche (SFTG Recherche), 75013 Paris, France; 7 Institut de Recherche Santé et Société, Université Catholique de Louvain, 1200 Bruxelles, Belgium

**Keywords:** social determinants of health, primary healthcare, integration

## Abstract

This paper aims to identify challenges and opportunities related to the integration of social determinants of health (SDH) into primary healthcare at an international symposium in Orford, Quebec, Canada. A descriptive qualitative approach was conducted. Three focus groups on different topics were led by international facilitators. Two research team members took notes during the focus groups. All the material was analyzed using a thematic analysis according to an inductive method. Many challenges were identified, leading to the identification of potential opportunities: integrate the concept of SDH in all phases of the training curriculum for health professionals to foster interprofessional and intersectoral collaboration and sociocultural skills; organize healthcare for better outreach to vulnerable populations; organize local and regional committees to develop management frameworks to produce and use territory-specific data; develop dashboards for primary healthcare providers describing the composition of their territory’s population; work collaboratively, rallying primary healthcare providers, community organization delegates, patient partners, citizens, and municipality representatives around common projects. Discussions prompted new directions for further primary healthcare research, among which are building on best practices in the literature and in the field, and engaging various stakeholders in research, including vulnerable populations, while focusing on patient experience.

## 1. Introduction

According to the World Health Organization, the social determinants of health (SDH) are defined as “the non-medical factors that influence health outcomes. They are the conditions in which people are born, grow, work, live, and age, and the wider set of forces and systems shaping the conditions of daily life. These forces and systems include economic policies and systems, development agendas, social norms, social policies and political systems” [1]. SDH interact with biological factors and behavior to impact on health [2]. Abundant literature demonstrates that SDH are associated with morbidity, mortality, and other health outcomes [3,4,5,6,7,8,9,10].

Among the ways to increase efficacy of care and health promotion in primary healthcare, greater consideration of SDH remains an important strategy [11,12]. DeVoe et al. (2016) [10] proposed a three-step framework to improve integration of SDH into primary healthcare practices: (1) collect and manage SDH data; (2) present and include SDH data in primary healthcare workflows; and (3) use SDH data to support action.

In a context where primary healthcare organizations worldwide are mobilized to improve integration of SDH, an international symposium gathered experts from the Groupe International Francophone de Soins Primaires [13] coming from France (YB, HF), Switzerland (CC, NS), Belgium (JM, TVD), and Quebec (CH, MB), and more than 100 various stakeholders to discuss these issues. The objective was to identify challenges and opportunities related to the integration of SDH into primary healthcare focusing on three topics: (1) strategies to integrate SDH in primary healthcare; (2) production and use of territory-specific data to identify at-risk populations; and (3) interaction between primary healthcare clinics and community organizations.

## 2. Materials and Methods

A qualitative descriptive approach [14] was used to identify the perceived challenges and the opportunities discussed during the symposium. Maximum variation sampling [15] (groups of participants) enabled organizers to gather more than 100 virtual and on-site participants, including primary healthcare researchers, decision-makers, managers, healthcare providers, patient partners, and trainees. Among the various stakeholders, 28 participated in one of the three on-site discussion groups of their choice. These groups focused on strategies to integrate SDH in primary healthcare, to use territory-specific data, and to foster interaction between primary healthcare clinics and community organizations, respectively. Some 10 participants participated in each group, comprising stakeholders from different membership groups and backgrounds. This participatory approach makes it possible to engage and integrate the views of a large group of stakeholders that are not necessarily considered by researchers [16]. All participants gave their verbal informed consent at the beginning of the symposium, after the research team presented the objectives of the event and the intention to publish the results. The Research Ethics Committee of the Centre intégré universitaire de santé et de services sociaux de l’Estrie-CHUS formally approved all procedures performed in this study, including the verbal informed consent.

Each discussion group was facilitated by an international expert following three questions: (1) What challenges do you perceive in relation to this topic? (2) What opportunities would you propose to overcome these challenges? (3) What are the priorities for the research agenda? Diversity within the groups aimed to promote exchanges that would help reveal a comprehensive portrait of the topic. One of the participants in each discussion group was assigned to take notes. Two members of the research team also took notes independently during the group discussions and the plenary session.

All notes and written reports of observations expressed as part of group discussions and plenary sessions were analyzed using a qualitative data analysis with an inductive approach. This type of analysis is well suited to identify central and obvious meanings, among the raw data, that are relevant to the research objectives [17]. To complete the analysis: (1) a research assistant prepared the raw data; (2) three authors read the data carefully and thoroughly; (3) one author identified the initial categories (initial coding); (4) during a research team meeting, all of the authors further reviewed, refined, and validated the categories [17]. Triangulation of sources (different groups of stakeholders from different professional disciplines and perspectives) and of researchers was used as a validation strategy [18,19].

## 3. Results

### 3.1. Strategies to Integrate SDH in Primary Healthcare

One of the main challenges raised by participants was that healthcare providers’ training remains focused on diseases and physical or mental health, but not enough on the SDH. As a result, providers may lack the knowledge and competency to evaluate and consider the SDH. In addition to practices being sparsely oriented towards SDH, this lack of expertise may increase the stigmatization of vulnerable populations.

Participants also noted challenges surrounding interprofessional collaboration and a tendency to work in silos. This issue adds to the difficulty as the combined expertise of different health and social care providers is often required to meet patients’ global needs. Stakeholders also noted that the current model of care is not always appropriate to meet the needs of low literacy or hard-to-reach populations. For these populations, interactions between primary healthcare and community organizations are particularly important (see Section 3.3).

To overcome these challenges, participants suggested better integration of the concept of SDH in all phases of the training curriculum for health professionals to foster interprofessional collaboration, and better explanations of “why” and “how” to do things. Stakeholders believed that the most vulnerable populations should have access to a team that works together and has access to communities of practice to improve their sociocultural skills. Primary healthcare should be organized in such a way as to improve the outreach to these vulnerable populations: services available directly in underprivileged environments; knowledge of available resources and opportunities; peer support, etc.

### 3.2. Production and Use of Territory-Specific Data to Identify at-Risk Populations

Participants explained that the existence of data is variable across countries, and they are often not organized to be easily accessed in practice, so they remain variable in nature and quality from one setting to another. In rare cases where these data are available, few people have the skills to interpret and use them. This is especially true for data about vulnerable populations who use fewer healthcare services. In many contexts, ethical and legal issues add barriers to data production and use.

As opportunities, stakeholders proposed that local and regional committees could develop management frameworks to produce and use data, by finding out what is available for a given territory, then planning ways of analyzing and interpreting these data. They also suggested developing more programs that integrate data into practice and creating dashboards for primary healthcare providers describing the composition of their territory’s population. Strategies to include data on the most vulnerable populations should be planned and the involvement of patient partners and citizens is an essential part of these reflections and actions.

### 3.3. Interaction between Primary Healthcare Clinics and Community Organizations

Participants explained that primary healthcare clinics and community organizations are two very different environments and that they suffer from the lack of a common vocabulary. Again, health professionals’ training does not prepare them to collaborate with members of community organizations. There is no shared standard process of reference and the gateway to collaboration is not always easy to find. Health professionals lack information about community organizations—and vice versa—and many confidentiality-related challenges arise when trying to work together. The value of community organizations was highlighted during certain stages of the COVID-19 pandemic, but it is not clear whether this really brought about any change.

Co-construction and consultation were perceived as necessary processes to foster this interaction. Working collaboratively, rallying primary healthcare providers, community organization delegates, patient partners, citizens, and even municipality representatives, around common projects could enable better understanding of each other’s reality and help to find opportunities that suit everyone. In the same way that training on interprofessional collaboration was considered necessary, training primary healthcare professionals to interact with community organizations was also suggested, as well as finding opportunities to maintain awareness of their territory’s community organization network (e.g., dashboards, interactive maps).

### 3.4. Next Steps for Research

Discussions surrounding these three key topics led participants to suggest additional directions for further primary healthcare research. Instead of reinventing the wheel, building on best practices was perceived as an important first step. Synthesizing best practices found in the literature and describing promising initiatives in the field were two avenues, keeping in mind that disseminating these best practices remains a crucial step.

Engaging various key stakeholders in research, including patient partners and citizens at the local level, emerged as a priority, as well as focusing on their experience (patient-reported experience or outcome measures, PREMs and PROMs). This engagement could help find ways of producing and using relevant data while finding the balance regarding the confidentiality and rights of data owners.

Inclusion of vulnerable populations in research teams and processes was also outlined as a major aim, to better understand their experience but also to find ways of offering them better care. In this sense, the expertise of community organizations could be helpful considering their level of interaction with this population.

## 4. Discussion

This international symposium highlighted challenges and opportunities related to the integration of SDH into primary healthcare, from the perspective of various stakeholders, including that of experts from four countries. The need for better integration of SDH in healthcare professionals’ curricula was outlined as an important first step. In their scoping review conducted to understand the current curricula designed to teach primary healthcare residents about SDH, Gard et al. (2019) [20] identified wide variation in curriculum development, implementation, and evaluation. Their review highlighted the need not only for systematic, consistent approaches to developing and delivering SDH curricula, but also for developing proper assessment of the curricula, especially the effects on learners’ behavior [20].

The WHO (2020) [21] recently conducted a systematic review, including 17 articles, to evaluate how primary healthcare organizations assess and consider SDH in their local population. They found that surveys and interviews were more commonly used to evaluate SDH than population-level data. The actions to address SDH ranged from individual-level interventions to population-wide measures and proposing representation of primary healthcare organizations on system-level policy and planning committees. They concluded that more observational and experimental evidence was needed to know if measuring SDH leads to interventions which reduce health disparities [21]. Another review by Boch et al. (2020) concluded that more research was needed on the best strategies for providers to screen for SDH in addition to how these practices influence resource referrals and use, as well as health improvements [22]. Pinto & Bloch advance that every broad primary healthcare organization should create an SDH committee to identify and support approaches to addressing SDH in a practical manner [23].

Andermann (2016) [24] proposed a framework for health providers to take action on the SDH in clinical practice, suggesting multi-level strategies, in line with our results. At the patient level, the framework suggests asking patients about social issues in a caring way and referring and helping them access appropriate resources. At the practice level, it proposes improving access and quality of care for hard-to-reach populations and integrating social support navigators into primary healthcare. At the community level, it suggests developing partnerships with community groups and local leaders, participating in community needs assessment, and engaging the community towards empowerment [24]. The French National College of General Practice produced recommendations for systematically identifying patients’ SDH, recording them in medical records in a standardized way, and acting on the basis of the identified determinants [25].

Building on the strategies used by organizations to develop SDH screening tools for ambulatory care and the barriers they faced, LaForge et al. (2018) [26] provided suggestions for others wanting to develop similar tools, and recommendations for further research. They conclude that development of SDH tools must consider that populations might have comparable data albeit tailored to local requirements. More research is still needed to determine how to implement these strategies in different care settings and how to use SDH data once collected [26].

This study has some limitations. A clear recording of discussions would have been difficult to obtain due to many discussions taking place in the same room. Therefore, the analysis was done using the discussion notes instead of a verbatim transcription. However, the triangulation of evaluators (diversity of viewpoints) as well as the validation steps helped improve the trustworthiness of results. The focus groups did not elaborate on the management framework mentioned in the Results section. Future studies could focus on the development of this kind of framework. Finally, although data collection was conducted in Quebec, a predominantly French-speaking Canadian province, most of the challenges and opportunities that were identified are transferable to other Canadian jurisdictions and other countries with similar public healthcare systems, i.e., a nationally legislated universal public health system in which provinces are responsible for organizing their own health services [27,28,29].

## 5. Conclusions

Integration of SDH into primary healthcare remains a priority for organizations worldwide, calling for collaborative and intersectoral efforts to improve curricula, screening of SDH and action at individual and populational levels, quality of care for hard-to-reach populations, and partnership with community organizations. Research questions were raised which will require participatory designs focusing on patients’ and citizens’ experience.

## Data Availability

The data presented in this study are available on request from the corresponding author.
